# Chemical Composition and Preliminary Screening of Anticholinesterase and Antioxidant Activities of the Essential Oil of *Ambrosia arborescens* Mill. from Southern Ecuador

**DOI:** 10.3390/plants15101447

**Published:** 2026-05-09

**Authors:** James Calva, Jorge Ramírez

**Affiliations:** Departamento de Química, Facultad de Ciencias Exactas y Naturales, Universidad Técnica Particular de Loja, San Cayetano Alto s/n, Loja 110107, Ecuador; jwcalva@utpl.edu.ec

**Keywords:** *Ambrosia arborescens*, AChE, γ-curcumene, antioxidant activity, chemical composition

## Abstract

*Ambrosia arborescens* Mill., a native medicinal plant traditionally used in the Andean region, has a poorly characterized essential oil (EO), with no prior reports on its anticholinesterase or antioxidant potential. As a first report and preliminary screening study, this work characterizes the chemical composition of the EO and evaluates its acetylcholinesterase (AChE) inhibitory and antioxidant activities. The EO was isolated by hydrodistillation and analyzed using gas chromatography coupled with mass spectrometry (GC–MS) and flame ionization detection (GC-FID). The biological activities were evaluated using the Ellman method to determine AChE inhibition and using ABTS and DPPH assays to determine antioxidant activity. Analysis of the chemical composition revealed 31 compounds, and the major components were γ-curcumene (28.63%), *trans*-muurola-4(14),5-diene (27.85%), and eucavone (18.46%). The EO showed moderate AChE inhibitory activity, with an IC_50_ value of 28.04 ± 1.02 µg/mL, and limited antioxidant activity, with ABTS SC_50_ = 373.75 ± 1.30 µg/mL and DPPH SC_50_ = 1101.84 ± 1.63 µg/mL. These findings demonstrate that the EO possesses selective anticholinesterase activity and limited antioxidant capacity. Given the structural diversity of its constituents, the observed bioactivity is likely the result of the combined contributions of multiple components; however, the specific active constituents and potential synergistic interactions require further investigation through bioassay-guided fractionation. These findings represent the first preliminary screening of the biological activities of *A. arborescens* EO and provide a foundation for future bioactivity-guided investigations.

## 1. Introduction

Essential oil derived from aromatic and medicinal plants is recognized as a rich source of bioactive secondary metabolites with diverse pharmacological properties [1,2], including antioxidant, anti-inflammatory, antimicrobial, and neuroprotective activities [3,4]. Controlling oxidative stress and supporting cholinergic function are especially important in neurodegenerative diseases like Alzheimer’s [5]. Current pharmacotherapies for neurodegenerative diseases present limited efficacy and are frequently associated with adverse effects. Natural substances with antioxidant activity and the ability to inhibit acetylcholinesterase (AChE) may offer new and safer options for therapy [6].

*Ambrosia arborescens* Mill. (Asteraceae), commonly known as “Marco”, “Altamisa”, “Santa María” or “Matico cimarron”, is a perennial shrub native to the Andean regions of South America [7]. It has been traditionally used in folk medicine as an anti-inflammatory, anti-tussive, anti-rheumatic, and anti-diarrheal agent [8]; for culture-bound “Espanto” syndromes; to treat disorders of the dermatological system [7,9,10], and in rheumatism and postpartum bathing [10]. Despite its ethnobotanical relevance, the phytochemical and pharmacological characterization of its essential oil remains underexplored. Previous phytochemical studies on *A. arborescens* have focused primarily on its non-volatile fraction, reporting the isolation of sesquiterpene lactones including damsin, coronopilin, psilostachyin, and psilostachyin C from the aerial parts and leaves of the plant [11], and the anticarcinogenic activity of these lactones and some semi-synthetic derivatives has also been evaluated against breast cancer cell lines, especially against cancer stem cells (CSCs) [12]. Comprehensive analyses of its volatile fraction, particularly about its neuropharmacological potential, have been scarce and fragmented.

The chemical composition of EO is highly variable and depends on factors such as geographic origin, harvest season, and extraction method [13,14]. In the genus *Ambrosia*, the volatile components are often dominated by monoterpenes and sesquiterpenes, but they vary greatly depending on the species examined and geographical location [15,16,17,18]. For instance, the EO of *A. artemisiifolia* was dominated by sesquiterpenes and monoterpenes such as germacrene D (25.3%), limonene (21.6%), and α-pinene (15.7%), which showed strong antibacterial activity [19]. The extract of *A. arborescens* from Peru showed antioxidant activity, and the activity of the ethyl lactate extract was a little higher than the methanol extract in terms of ORAC (715.38 ± 3.2) and DPPH (263.04 ± 2.8) [20]. Similarly, a recent characterization of *A. arborescens* EO from Peru identified sesquiterpenes as the predominant class, with germacrene D (16.54%), santolina triene (14.3%), and ar-curcumene (5.17%) as notable constituents, confirming the intraspecific chemical variability of this species across Andean populations [21]. Another study in the same species showed that it was rich in β-acoradiene (15.3%) and chrysanthemone (11.3%) [22]. Previous studies have demonstrated potential applications in pest control as a natural insecticide, as an attractant or repellent for *Thrips tabaci*, and in medicine as a bactericide against *Bacillus subtilis* and *Bacillus cereus*, and as an anthelmintic against *Aedes aegypti* [21,22,23,24,25].

To date, no peer-reviewed study has reported the acetylcholinesterase inhibitory or antioxidant activity of the EO of *A. arborescens*, representing a significant gap in the phytopharmacological characterization of this Andean species. The present work is therefore conceived as a first report and preliminary screening study aimed at generating baseline bioactivity data that can guide future mechanistic and bioassay-directed investigations.

Given the rising interest in plant-derived multi-functional agents for neurodegenerative diseases, this preliminary screening study aims to (i) characterize the chemical composition of the essential oil of *A. arborescens* collected from the Ecuadorian Andes using gas chromatography coupled with mass spectrometry (GC–MS) and flame ionization detection (GC-FID) and (ii) provide the first report of its acetylcholinesterase inhibitory and antioxidant activities as an initial step toward its pharmacological valorization. The findings contribute to the scientific validation of traditional uses of *A. arborescens* and may identify specific volatile constituents responsible for its dual bioactivity, thereby supporting its potential as a source of neuroprotective phytochemicals.

## 2. Results

### 2.1. Chemical Analysis

**Figure 1:**
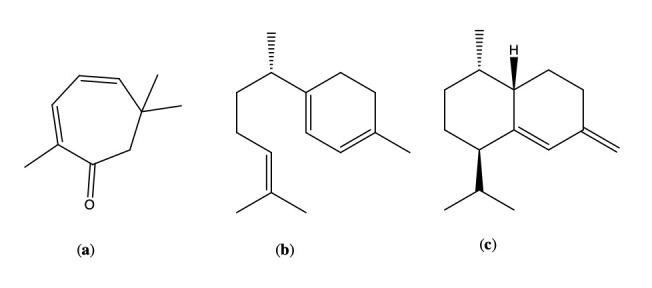
Majority compounds of *Ambrosia arborescens* essential oil: (**a**) eucarvone, (**b**) γ-curcumene, and (**c**) *trans*-muurola-4(14),5-diene.

**Figure 2:**
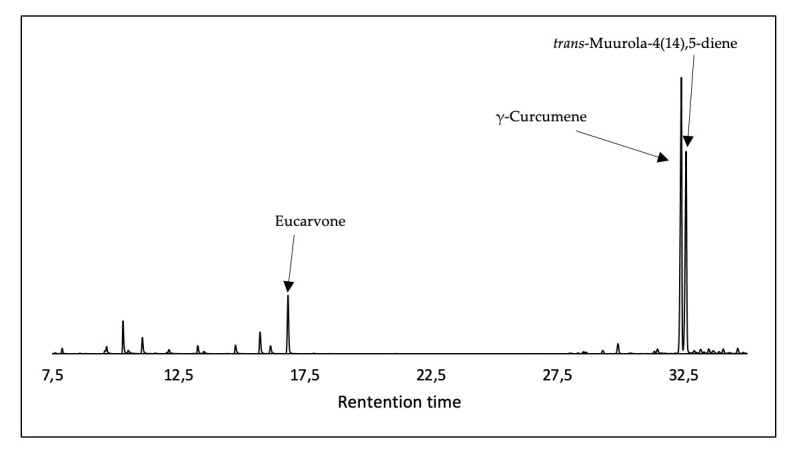
Chromatogram of *Ambrosia arborescens* essential oil collected in Ecuador.

The EO distillation yielded 1.09 ± 0.10% (*w*/*w*). In the chemical analysis of the EO of *A. arborescens*, a total of thirty-one compounds were identified and estimated by peak area normalization, representing 98.62% of the total EO. The EO was mainly composed of hydrocarbons and sesquiterpenes, corresponding to 63.18% of the total composition. The major compounds in Figure 1 were γ-curcumene (28.63 ± 0.63%), *trans*-muurola-4(14),5-diene (27.85 ± 0.47%), and the monoterpene eucarvone (18.46 ± 0.70%). The chemical composition of the EO is shown in Table 1 and Figure 2, according to the elution order in the DB-5MS column.

### 2.2. Cholinesterase Inhibitory Activity

**Figure 3:**
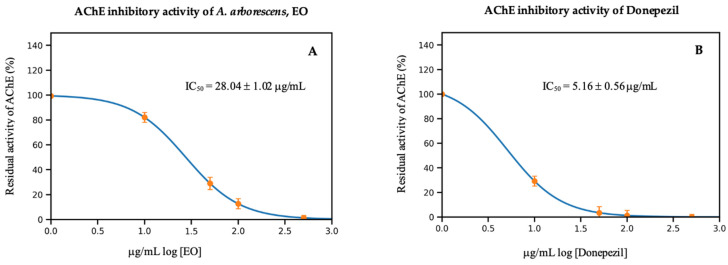
Dose-dependent inhibitory effect of *Ambrosia arborescens* essential oil (**A**) and donepezil (**B**) on acetylcholinesterase (AChE) activity. The results demonstrate a progressive increase in inhibition with rising concentrations, suggesting a potential interaction between oil constituents and the enzyme. Values are expressed as mean ± standard deviation (*n* = 3).

The EO from *A. arborescens* leaves was used to see how well it could block the AChE enzyme. In this preliminary screening, the EO of *A. arborescens* exhibited moderate AChE inhibitory activity, with an IC_50_ value of 28.04 ± 1.02 µg/mL, indicating concentration-dependent inhibition that warrants further mechanistic characterization. Under identical assay conditions, donepezil showed an IC_50_ of 5.16 ± 0.56 µg/mL (see Figure 3). The inhibitory activity of *A. arborescens* EO has not been previously reported. Considering that the essential oils (EOs) are a complex multi-component mixture rather than a single pure compound, this level of inhibitory activity is pharmacologically relevant and supports further bioactivity-guided investigation aimed at identifying the specific constituent(s) responsible for the observed effect.

### 2.3. Antioxidant Activity

The antioxidant activity of the essential oil of *A. arborescens* was evaluated using the ABTS radical scavenging assay. The results were compared to those of Trolox, a water-soluble vitamin E analog widely used as a reference standard due to its well-characterized and reproducible radical-scavenging activity (Table 2). The essential oil exhibited moderate antioxidant activity, with a SC_50_ value substantially higher (i.e., lower potency) than that of Trolox. Notably, eucarvone, the major constituent identified in the EO, has not been previously associated with significant ABTS scavenging activity, suggesting that the observed antioxidant effect cannot be attributed to any single dominant constituent based on their known chemical properties. Future studies employing bioassay-guided fractionation are necessary to identify the constituent(s) responsible for this activity.

## 3. Discussion

Phytochemical analysis revealed that the EO of *A. arborescens* collected in southern Ecuador contained three major components, which were γ-curcumene (28.63 ± 0.63%), *trans*-muurola-4(14),5-diene (27.85 ± 0.47) and eucarvone (18.46 ± 0.70). This profile differs from previously reported compositions of the same species. Ruiz et al. characterized the EO of *A. arborescens* from Peru and identified germacrene D (36.96%) and β-himachalene (30.62%) as the predominant constituents [24], a sesquiterpene-hydrocarbon-dominated profile that, while sharing the same dominant chemical class as our findings, differs substantially at the compound level [22]. More recently, Solís-Quispe et al. identified 113 compounds in the EO of *A. arborescens* from the Peruvian Andes, representing 94.7% of the total composition [22], with β-himachalene, germacrene D, and α-bisabolol among the major constituents [20]. A further characterization of *A. arborescens* EO from Peru reported germacrene D (16.54%), santolina triene (14.3%), and ar-curcumene (5.17%) as notable constituents, with eucarvone also detected at lower percentages [21]. This differentiation may be due to the collection site or environmental conditions, which can significantly influence the composition [14,27].

The EO characterized in this study represents a distinct sesquiterpene hydrocarbon chemotype, defined by the co-dominance of γ-curcumene and *trans*-muurola-4(14),5-diene, with eucarvone as the principal oxygenated component. To the best of our knowledge, this chemotype has not been previously described for *A. arborescens* and differs fundamentally from all reported chemotypes of the species, which are consistently defined by thujone, camphor, or chamazulene dominance. The emergence of this chemotype in the Ecuadorian Andes is consistent with the biosynthetic plasticity documented by Barra [13] and with the findings of Calva et al. [14] and Matzrafi et al. [17], who demonstrated that intraspecific variation in *Ambrosia* volatile profiles is strongly modulated by altitude, phenological stage, and edaphoclimatic conditions.

The EO of *A. arborescens* exhibited moderate AChE inhibitory activity, with an IC_50_ of 28.04 ± 1.02 µg/mL. γ-Curcumene, the most abundant constituent (28.63%), is a bisabolane-type sesquiterpene whose structural analogs have been implicated in AChE inhibition through non-covalent interaction with the peripheral anionic site (PAS) and the catalytic active site (CAS) of the enzyme [28,29], *trans*-muurola-4(14),5-diene (27.85%), a bicyclic sesquiterpene of the muurolane skeleton, has not been directly studied for AChE inhibition; however, its rigid bicyclic framework and calculated lipophilicity (log P > 4) suggest a capacity for passive penetration into the enzyme active site gorge, which is predominantly hydrophobic in character [29]. Eucarvone (18.46%), a bicyclic oxygenated monoterpene (an isopropenyl cyclohexenone), has been reported to lack significant radical scavenging activity, but its α,β-unsaturated carbonyl functionality could in principle participate in reversible covalent or electrostatic interactions with nucleophilic residues at the AChE active site, a mechanism analogous to that proposed for enone-bearing natural products [30,31]. Nevertheless, it is important to recognize that the AChE inhibitory activity observed cannot be attributed to any single constituent in isolation. Essential oils act as multi-component systems in which synergistic and additive interactions among constituents frequently determine the overall bioactivity.

The aim of this study was to investigate the inhibitory effect of *A. arborescens* EO on acetylcholinesterase. This enzyme plays a central role in cholinergic neurotransmission and constitutes a validated therapeutic target of neurodegenerative disorders, like Alzheimer’s disease. Madar et al. [5] highlight the urgency of finding new therapeutic targets for the treatment of Alzheimer’s, while Ribaudo et al. [6] emphasize the importance of nature-inspired compounds that can act as dual inhibitors in the neurodegenerative pathway.

The inhibitory activity observed in our samples may be attributed to specific constituents such as monoterpenes or sesquiterpenes. According to De Sousa et al. [2], essential oil constituents serve as an alternative source for novel antidepressants and neuro-active agents. In fact, some studies conducted on essential oils from plants such as *Lippia origanoides*, *Piper crassipes*, *Lepisanthes rubiginosa*, *Pseuduvaria macrophylla*, *Pavetta graciliflora*, *Syzygium variolosum*, and *Aloysia triphylla* exhibit varying degrees of anticholinesterase activity [32,33,34,35,36,37,38]. Essential oils, such as those from *Lippia origanoides* and *Piper crassipes*, have demonstrated significant acetylcholinesterase (AChE) inhibitory activity [31,32,33,34]. Meanwhile, species such as *Pseuduvaria macrophylla* and *Pavetta graciliflora* exhibited weaker inhibitory activity [35,36].

The limited antioxidant activity of *A. arborescens* EO (ABTS SC_50_ = 373.75 ± 1.30 µg/mL; DPPH SC_50_ = 1101.84 ± 1.63 µg/mL) is directly attributable to the structural composition of its dominant constituents. Both DPPH and ABTS assays operate primarily through hydrogen-atom transfer (HAT) and single-electron transfer (SET) mechanisms, which require labile O–H moieties with low bond dissociation energies—structural features characteristic of phenolic compounds [39,40]. This interpretation is consistent with the well-established observation that the antioxidant activity of essential oils correlates directly with their phenolic content, and that monoterpene and sesquiterpene hydrocarbons contribute negligible radical-scavenging activity [41,42]. The mechanism of inhibition (competitive, noncompetitive, or uncompetitive) was not determined in this study and warrants further kinetic characterization. These antioxidant activities are important because they help neutralize reactive oxygen species (ROS), which play a role in cancer development [8] and in the spoilage of food [3]. Compared to other species in the same genus, *A. arborescens* has some special characteristics. For example, *Ambrosia trifida* is known for its antimicrobial and plant-inhibiting effects [15,18], while *Ambrosia artemisiifolia* stands out for its ability to fight certain plant diseases [19]. However, *A. arborescens* seems especially promising for medicinal use, a fact supported by its long history in traditional medicine in places like Loja and Zamora-Chinchipe, Ecuador [10].

As a first report and preliminary screening study, the present work contributes foundational bioactivity data for *A. arborescens* EO to the growing body of evidence on anticholinesterase activity in essential oils from Ecuadorian and Andean flora, including *Aloysia triphylla*, *Clinopodium taxifolium*, *Lepechinia paniculata*, *Piper arboreum*, *Clinopodium brownei*, *Salvia pichinchensis*, *Diplosthephium juniperinum*, and *Bixa orellana*, [38,43,44,45,46,47,48,49]. The present study is the first to document this activity for *A. arborescens* EO and identifies a chemotypically distinct Ecuadorian accession of this species with a selective anticholinesterase profile, thereby expanding the pharmacological landscape of neotropical biodiversity and supporting the basis for future bioassay-guided isolation studies.

## 4. Materials and Methods

### 4.1. Plant Material

The collection of *A. arborescens* took place during the late flowering stage in the parish of San Lucas, Moraspamba neighborhood, coordinates 3°43′52″ S, 79°16′7″ W, in the province of Loja, Ecuador. The plant collection was carried out under permit by the Ministry of Environment, Water and Ecological Transition (MAATE), Number MAATE-DBI-CM-2022-0248. The botanical specimen was identified by Dr. Fani Tinitana, curator at the herbaria of the Universidad Técnica Particular de Loja (HUTPL). The voucher has been deposited in the HUTPL, Ecuador, under code FT045MCAT. The plant material was subjected to steam distillation immediately after collection.

### 4.2. Distillation of the Volatile Fraction

The fresh aerial parts of *A. arborescens* (600 g) were manually fragmented with scissors until uniform-sized sections were obtained and placed in a 2 L reaction flask. The distillation was performed for 4 h. Once the process was complete, the organic phase was separated, dried over anhydrous sodium sulfate (Na_2_SO_4_), filtered, and weighed to determine the yield. The EO obtained was stored at −4 °C until further analysis in order to preserve its chemical and physical properties. All distillations were performed in triplicate to ensure the reproducibility of the results.

### 4.3. Qualitative Analysis of the EO

Qualitative analysis of EO was performed by a GC-MS Thermo Scientific Trace 3000 device coupled to a Mass Spectrum ISQ 7200 (Thermo Fisher Scientific, Waltham, MA, USA), by injecting 1 µL of each distilled fraction, diluted 1% (*v*/*v*) in dichloromethane. The injector was kept at 220 °C and operated in split mode with a split ratio of 40:1. The carrier gas (He) was set at a constant flow of 1 mL/min. The analysis was performed under thermal gradient conditions with the following temperature program 60 °C for 5 min, increased to 110 °C at a rate of 5 °C/min, then to 148 °C at a rate of 2 °C/min and to 250 °C at a rate of 20 °C/min, then held at 250 °C for 2.4 min. The MS was operated in the SCAN mode with a scan rate of 2 scans/s within a mass range of 40–350 m/z at 70 eV [48]. For each chromatographic peak, the corresponding linear retention index (LRI) was calculated according to van den Dool and Kratz [50], with reference [26] to a mixture of a homologous series of n-alkanes C10-C25 (TPH-6RPM of CHEM SERVICE), analyzed by GC under the same conditions as the essential oil. A non-polar capillary column, DB-5MS (5% phenyl-methylpolysiloxane, 30 m × 0.25 mm, 0.25 μm film thickness), was used. The procedure was repeated in triplicate.

### 4.4. Quantitative Analysis of the EO

The quantitative analysis of the EO was performed using gas chromatography coupled to a flame ionization detector (GC-FID). We employed the same instrumentation and chromatographic conditions as in the qualitative GC-MS analysis. All analytical parameters were identical to those described for the GC-MS analysis to ensure methodological consistency. The relative percentages reported represent peak area normalization values obtained by GC-FID and should be considered as semi-quantitative estimates. In the absence of calibration curves or application of FID response correction factors, the absolute quantification of individual components cannot be guaranteed, particularly for chemically diverse constituents such as oxygenated monoterpenes and sesquiterpene hydrocarbons. This limitation is acknowledged and does not affect the qualitative identification of constituents. The results are expressed as mean values ± standard deviations based on three independent injections.

### 4.5. AChE Inhibitory Activity

The inhibitory activity of *A. arborescens* EO against acetylcholinesterase was evaluated spectrophotometrically using the modified Ellman method [51]. A stock solution of the essential oil was prepared by dissolving 10 µL in 990 µL of methanol (10 mg/mL), followed by serial two-fold dilutions to yield final test concentrations of 8000, 4000, 2000, 1000, 500, 250, 125, and 62.5 µg/mL. The reaction mixture in each well of a 96-well microplate consisted of 60 µL of phosphate-buffered saline (PBS, pH 7.4), 20 µL of acetylthiocholine iodide (ATCh, 15 mM), 100 µL of 5,5′-dithiobis-(2-nitrobenzoic acid) (DTNB, 0.3 mM in 0.1 M Tris-HCl buffer, pH 8.0), and 20 µL of the diluted essential oil or control. After pre-incubation at 25 °C for 3 min with gentle shaking, the enzymatic reaction was initiated by adding 20 µL of AChE solution (0.5 U/mL in PBS). The hydrolysis of ATCh was monitored kinetically for 60 min at 405 nm using a BioTek EPOCH 2 microplate reader (BioTek, Winooski, VT, USA).

The half-maximal inhibitory concentration (IC_50_, µg/mL) was determined by nonlinear regression analysis using the four-parameter sigmoidal dose–response model (variable slope) implemented in GraphPad Prism v8.0.1 (GraphPad Software, San Diego, CA, USA), according to the Equation (1):

(1) Y=Bottom+(Top−Bottom)[1+10^(LogIC50−X×HillSlope)]

where: *Y* is the percentage of AChE inhibition, *X* is the logarithm of the inhibitor concentration (log µg/mL), *Top* and *Bottom* are the upper and lower plateaus of the curve (constrained to 100 and 0, respectively), *LogIC*_50_ is the logarithm of the concentration producing 50% inhibition, and *HillSlope* is the Hill coefficient reflecting the steepness of the dose–response curve [52,53]. The Hill slope obtained for the EO was 1.8 ± 0.3, which suggests positive cooperativity or multi-site interactions, indicating that the inhibitory effect intensifies steeply within a narrow concentration range.

### 4.6. Antioxidant Activity of EO

The antioxidant activity of *A. arborescens* essential oil was evaluated using two widely validated spectrophotometric assays: DPPH (2,2-diphenyl-1-picrylhydrazyl) free radical scavenging and ABTS (2,2′-azino-bis(3-ethylbenzothiazoline-6-sulfonic acid)) radical cation reduction. Both assays were performed in triplicate, and the results are expressed as 50% scavenging concentration (SC_50_, µg/mL), determined by nonlinear regression analysis using the four-parameter sigmoidal dose–response model (variable slope) in GraphPad Prism v8.0.1 (GraphPad Software, San Diego, CA, USA), according to Equation (2):

(2) Y=Bottom+(Top−Bottom)[1+10^(LogSC50−X×HillSlope)]

where *Y* is the percentage of radical scavenging activity (%), *X* is the logarithm of the sample concentration (log µg/mL), and *HillSlope* is the Hill coefficient [52].

For the DPPH assay, a stock solution was prepared by dissolving 4.6 mg of DPPH in 100 mL of HPLC-grade methanol. This solution was adjusted with methanol to obtain an absorbance of 1.10 ± 0.02 at 515 nm, measured in an EPOCH 2 microplate reader (BioTek, Winooski, VT, USA). In 96-well plates, 270 µL of the adjusted DPPH solution and 30 µL of the EO were previously diluted in methanol and added in a series of decreasing concentrations. The reaction was incubated at room temperature in the dark for 60 min, and the final absorbance was recorded at 515 nm. Trolox was used as a positive control, and methanol as a blank.

For the ABTS assay, the ABTS radical was generated by chemical oxidation when equal volumes of ABTS (7.4 mM) and potassium persulfate (2.6 mM) were mixed in ultrapure water. The mixture was stirred for 12 h at room temperature in the dark. The stock solution was then diluted with methanol to an absorbance of 1.10 ± 0.02 at 734 nm. In 96-well plates, 270 µL of the adjusted ABTS solution was combined with 30 µL of the EO (at the same concentrations as in the DPPH assay). The reaction was incubated for 60 min in the dark at room temperature, and the absorbance was measured at 734 nm. Trolox was used as the positive control, and methanol as the blank.

### 4.7. Statistical Analysis

All experimental determinations, including essential oil yield, chemical composition by GC-FID, AChE inhibitory activity, and antioxidant activity (DPPH and ABTS assays) were performed in biological and analytical triplicate (*n* = 3). Quantitative data are expressed as mean ± standard deviation (SD). The normality of the data distribution was assessed prior to inferential analysis. Differences among treatment groups were evaluated by one-way analysis of variance (ANOVA), followed by Tukey’s Honest Significant Difference (HSD) post-hoc test for multiple pairwise comparisons. Statistical significance was defined at *p* < 0.05. Half-maximal inhibitory concentration (IC_50_) and half-maximal scavenging concentration (SC_50_) values were determined by nonlinear regression analysis using a four-parameter sigmoidal dose–response model (log[inhibitor] vs. normalized response—variable slope) implemented in GraphPad Prism v8.0.1 (GraphPad Software, San Diego, CA, USA).

## 5. Conclusions

The essential oil isolated from the leaves of *A. arborescens* was analyzed, and 31 compounds were found, with γ-curcumene (28.63%), trans-muurola-4(14),5-diene (27.85%), and eucarvone (18.46%) as the dominant constituents. To the best of our knowledge, this is the first time that the biological tests showed that this oil could help to inhibit acetylcholinesterase (AChE) and has a moderate antioxidant activity, as seen in the ABTS assay. These findings indicate that the oil could have multiple uses, especially in research on neurodegenerative diseases. The preliminary screening of biological activity revealed moderate AChE inhibitory activity (IC_50_ = 28.04 ± 1.02 µg/mL) and limited antioxidant capacity (ABTS SC_50_ = 373.75 µg/mL; DPPH SC_50_ = 1101.84 µg/mL). These observed biological activities of the EO cannot be attributed to any single constituent based on the available experimental evidence. A key limitation of this study is the absence of bioassay-guided fractionation, which would allow the direct attribution of the observed anticholinesterase activity to specific constituents or fractions. Future work should include chromatographic separation of the EO, followed by individual testing of isolated fractions and major constituents (γ-curcumene, trans-muurola-4(14),5-diene, and eucarvone), as well as isobolographic or combination index analyses to evaluate potential synergistic interactions. This research highlights the potential of neotropical biodiversity and lays the groundwork for future scientific studies.

## Figures and Tables

**Table 1:** Chemical composition of *Ambrosia arborescens* Mill. essential oil from Ecuador.

N°	Compound	LRI_cal_^1^	LRI_lit_^2^	%±SD
1	Santolina triene	904	906	1.72 ± 0.15
2	α-Pinene	935	932	1.26 ± 0.16
3	Artemiseole	977	971	1.26 ± 0.16
4	β-Pinene	993	974	1.94 ± 0.27
5	ο-Cymene	1031	1022	2.02 ± 0.06
6	Limonene	1034	1024	0.68 ± 0.07
7	NI	1058	--	0.55 ± 0.03
8	Terpinolene	1091	1086	1.02 ± 0.01
9	2,6-Dimethylphenol	1111	1106	0.56 ± 0.03
10	Chrysanthenone	1120	1124	1.41 ± 0.04
11	Eucarvone	1135	1146	18.46 ± 0.70
12	*trans*-p-Mentha-8-thiol-3-one	1365	1371	1.33 ± 0.10
13	NI	1369	--	0.01 ± 0.01
14	NI	1388	--	0.39 ± 0.01
15	NI	1393	--	0.01 ± 0.01
16	β-Elemene	1395	1389	0.87 ± 0.03
17	α-Cedrene	1411	1410	1.21 ± 0.05
18	(*E*)-Caryophyllene	1426	1417	1.46 ± 0.07
19	Geranyl acetone	1459	1453	0.99 ± 0.07
20	Linalool isovalerate	1463	1466	0.47 ± 0.07
21	γ-Gurjunene	1468	1475	0.05 ± 0.01
22	γ-Curcumene	1484	1481	28.63 ± 0.63
23	*trans*-Muurola-4(14),5-diene	1490	1493	27.85 ± 0.47
24	γ-Patchoulene	1505	1502	0.61 ± 0.03
25	(*E*,*E*)-α-Farnesene	1510	1505	1.38 ± 0.05
26	(*Z*)-γ-Bisabolene	1517	1514	0.37 ± 0.01
27	δ-Cadinene	1526	1522	0.74 ± 0.05
28	1,10-di-epi-Cubenol	1617	1618	1.06 ± 0.07
29	NI	1797	--	0.05 ± 0.01
30	NI	1802	--	0.41 ± 0.05
31	(6*Z*,10*E*)-Pseudo phytol	2042	2030	1.09 ± 0.09
	Hydrocarbon monoterpenes			10.05%
	Oxygenated monoterpenes			21.05%
	Hydrocarbon sesquiterpenes			63.18%
	Oxygenated sesquiterpenes			1.54%
	Other			2.08%
	Total identified			98.62%

^1^ LRI_cal_ Calculated linear retention index; ^2^ LRI_lit_ Linear retention index from Adams [26]; %±SD mean of relative percent area and standard deviation content in the EO over three determinations, NI not identified compounds its mass spectra in Supplementary Material Figures S1–S6.

**Table 2:** Half scavenging capacity (SC_50_) of *A. arborescens* essential oil.

Sample	ABTS	DPPH
	SC_50_ ± SD	(µg/mL)
*A. arborescens* Mill. EO	373.75 ± 1.30	1101.84 ± 1.63
Trolox *	7.28 ± 0.26	8.90 ± 0.26

* Trolox was used as a positive reference, and values originally determined as 29.09 ± 1.05 µM (ABTS) and 35.54 ± 1.04 µM (DPPH), converted to µg/mL using MW = 250.29 g/mol (CAS 53188-07-1)

## Data Availability

Data supporting the results presented can be found throughout the article. Any additional information can be communicated to the corresponding author.

## Supplementary Materials

The following supporting information can be downloaded at: https://www.mdpi.com/article/10.3390/plants15101447/s1, Figure S1: Mass spectrum of the unidentified components. Peak unidentified 1: RT 14.53; Figure S2: Mass spectrum of the unidentified components. Peak unidentified 2: RT 29.08; Figure S3: Mass spectrum of the unidentified components. Peak unidentified 3: RT 29.82; Figure S4: Mass spectrum of the unidentified components. Peak unidentified 4: RT 30.24; Figure S5: Mass spectrum of the unidentified components. Peak unidentified 5: RT 46.00; Figure S6: Mass spectrum of the unidentified components. Peak unidentified 5: RT 46.00

## Abbreviations

The following abbreviations are used in this manuscript:

AChE	Acetylcholinesterase
EO	Essential oils
VOCs	Volatile organic compounds
DPPH	2,2-Diphenyl-1-picrylhydrazyl
ORAC	Oxygen radical absorbance capacity
ABTS	2,2′-Azino-bis(3-ethylbenzothiazoline-6-sulfonic acid)
