# Supplementary material for: Chemical Composition and Preliminary Screening of Anticholinesterase and Antioxidant Activities of the Essential Oil of Ambrosia arborescens Mill. from Southern Ecuador
Source: Plants (Basel). 2026 May 9;15(10):1447. doi: 10.3390/plants15101447 (PMC13210825; doi:10.3390/plants15101447)

plants

MDPI

Supplementary material

Chemical Composition, Anticholinesterase and Antioxidant Activities of the Essential Oil of *Ambrosia arborescens* Mill. from Southern Ecuador

James Calva [1] and Jorge Ramírez [1,]*

[1] Departamento de Química, Facultad de Ciencias Exactas y Naturales, Universidad Técnica Particular de Loja, San Cayetano Alto s/n, Loja 110107, Ecuador; jwcalva@utpl.edu.ec (J.C.); jyramirez@utpl.edu.ec (J.R.)
* Correspondence: jyramirez@utpl.edu.ec

Figure S1. Mass spectrum of the unidentified components. Peak unidentified 1: RT 14.53.

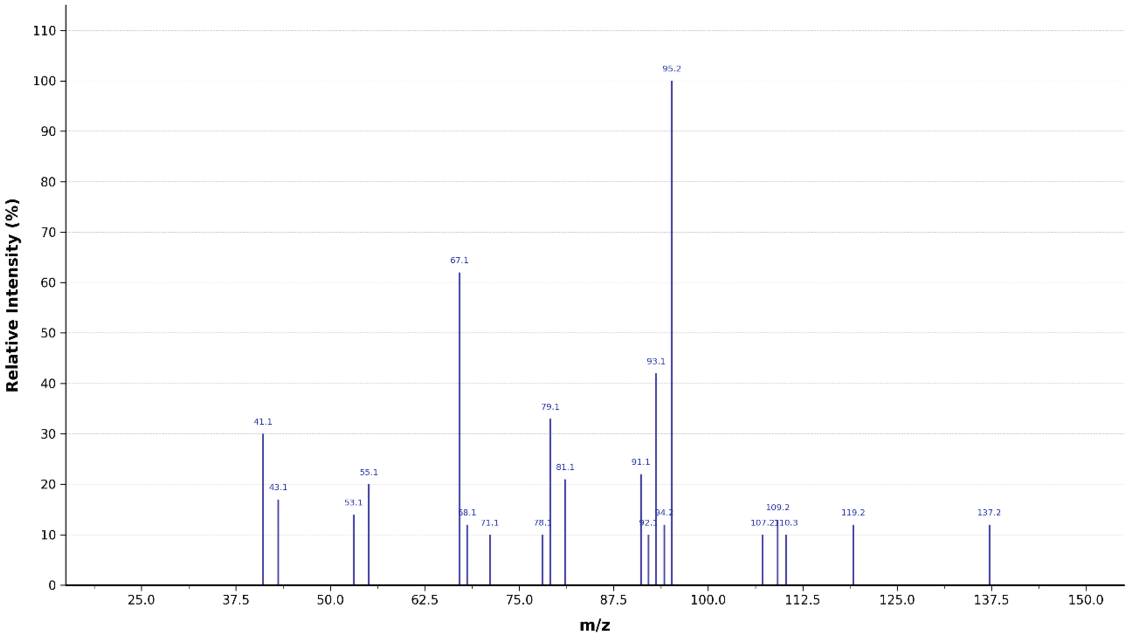

 https://doi.org/10.3390/xxxxx

Figure S2. Mass spectrum of the unidentified components. Peak unidentified 2: RT 29.08

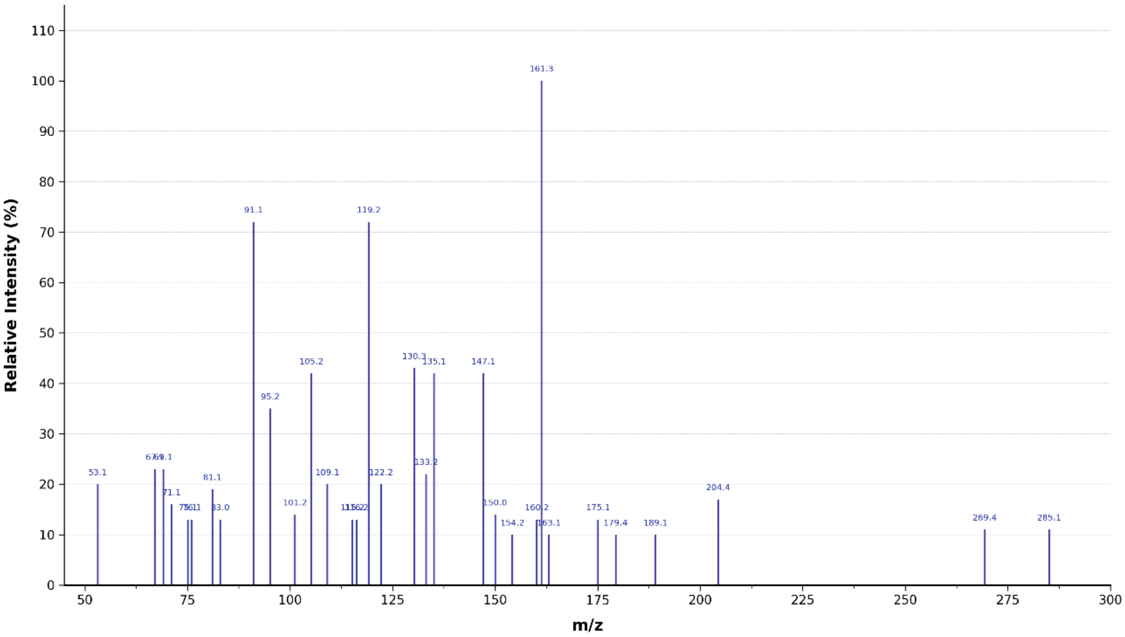

Figure S3. Mass spectrum of the unidentified components. Peak unidentified 3: RT 29.82

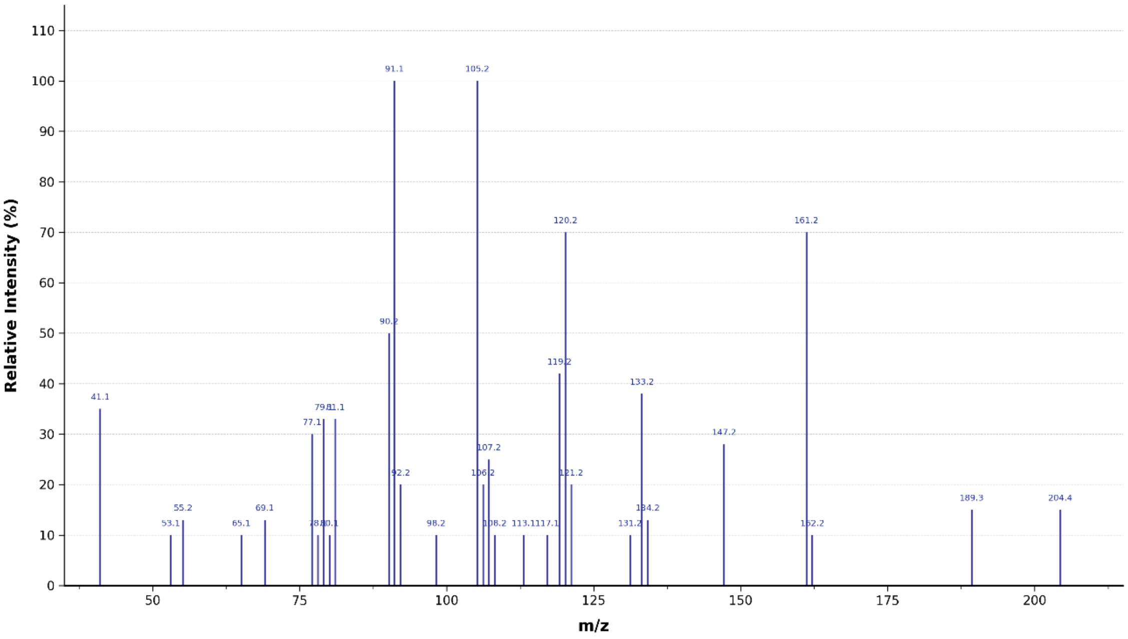

Figure S4. Mass spectrum of the unidentified components. Peak unidentified 4: RT 30.24

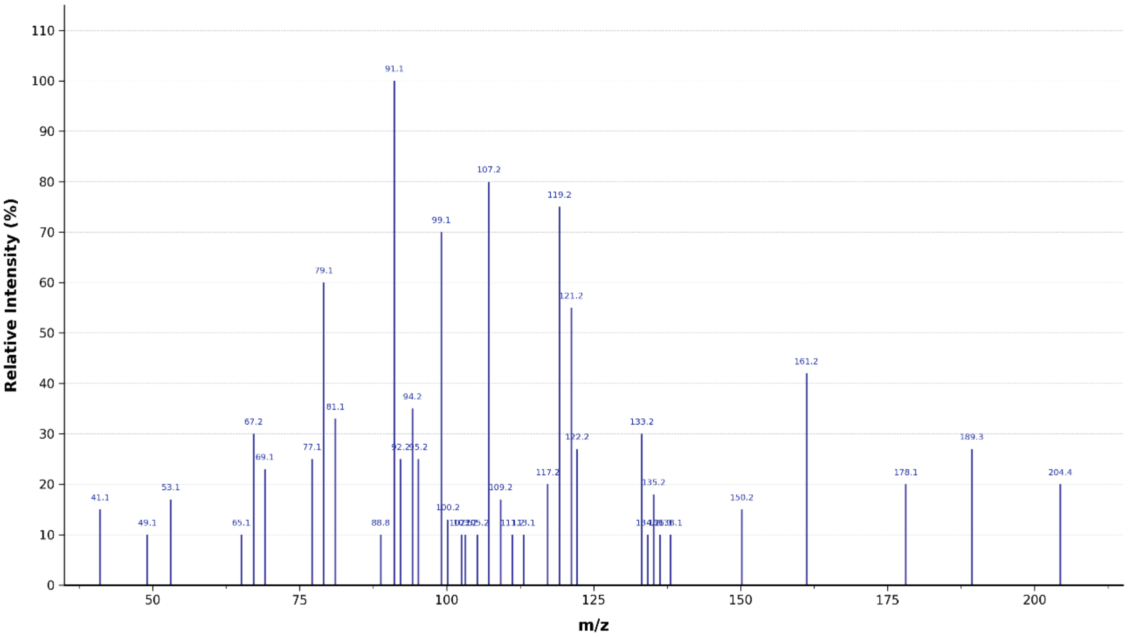

Figure S5. Mass spectrum of the unidentified components. Peak unidentified 5: RT 46.00

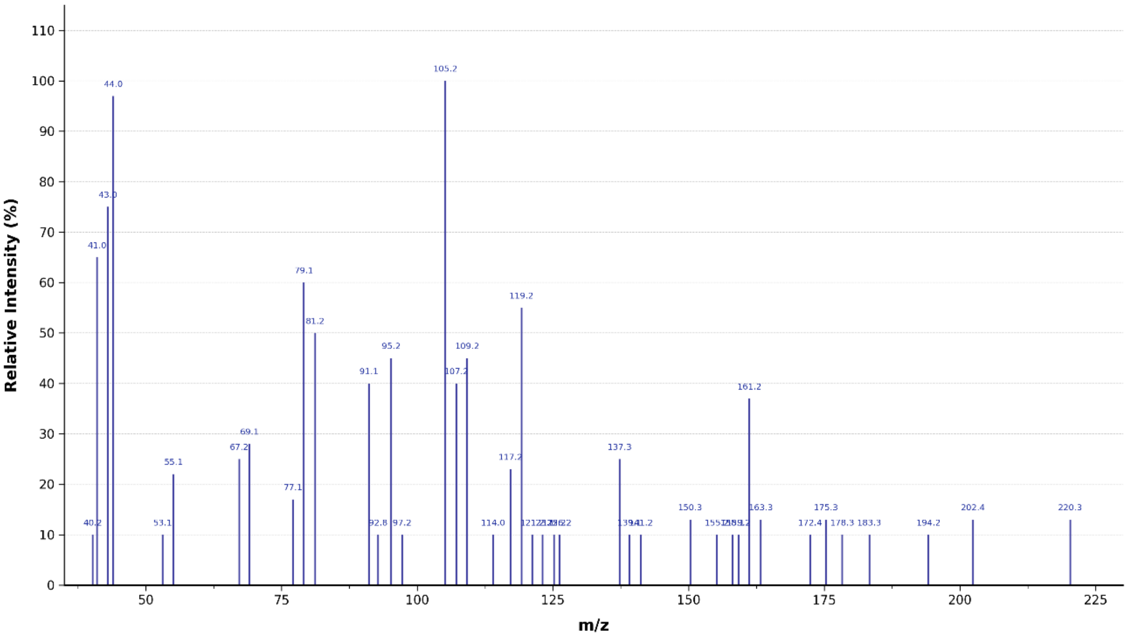

Figure S6. Mass spectrum of the unidentified components. Peak unidentified 6: RT 46.35

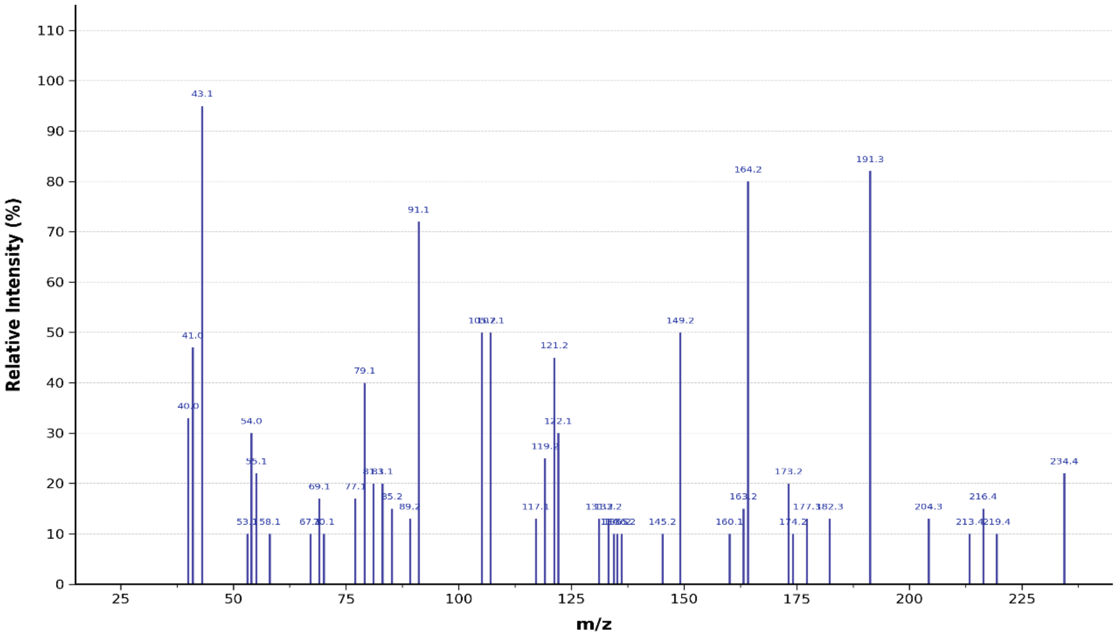

Supplement: Supplementary file 1 [file plants-15-01447-s001.zip › plants-4253392-supplementary.pdf]
